# Characterization and Inducing Melanoma Cell Apoptosis Activity of Mannosylerythritol Lipids-A Produced from *Pseudozyma aphidis*

**DOI:** 10.1371/journal.pone.0148198

**Published:** 2016-02-01

**Authors:** Linlin Fan, Hongji Li, Yongwu Niu, Qihe Chen

**Affiliations:** 1 College of Biosystems Engineering and Food Science, Zhejiang University, Hangzhou 310058, P. R. China; 2 Fuli Institute of Food Science, Zhejiang University, Hangzhou 310058, P. R. China; University of Manitoba, CANADA

## Abstract

Mannosylerythritol lipids (MELs) are natural glycolipid biosurfactants which have potential applications in the fields of food, cosmetic and medicine. In this study, MELs were produced from vegetable oil by *Pseudozyma aphidis*. Their structural data through LC/MS, GC/MS and NMR analysis revealed that MEL-A with two acetyls was the major compound and the identified homologs of MEL-A contained a length of C8 to C14 fatty acid chains. This glycolipid exhibited a surface tension of 27.69 mN/m at a critical micelle concentration (CMC), self-assembling into particles in the water solution. It was observed to induce cell growth-inhibition and apoptosis of B16 melanoma cells in a dose-dependent manner, as well as cause cell cycle arrest at the S phase. Further quantitative RT-PCR analysis and western blotting revealed an increasing tendency of both mRNA and protein expressions of Caspase-12, CHOP, GRP78 and Caspase-3, and a down-regulation of protein Bcl-2. Combined with the up regulation of signaling IRE1 and ATF6, it can be speculated that MEL-A-induced B16 melanoma cell apoptosis was associated with the endoplasmic reticulum stress (ERS).

## Introduction

Biosurfactants are mainly produced by a variety of microorganisms. These extracellular amphiphilic compounds exhibited advantages over the synthetic ones, including biodegradability, low toxicity, excellent surface-activity and stability [1, 2]. Their applications in the food, cosmetic and pharmaceutical and environmental industries have been proved in the previous studies.

Mannosylerythritol lipids (MELs) belong to the glycolipid biosurfactants. They contain polar moiety of 4-O-β-D-mannopyranosyl-D-erythritol and the non-polar hydrophobic fatty acid chains [3]. Their chemical structures vary with the different fatty acid chains and the acetyl groups, commonly known as MEL-A, MEL-B, MEL-C and MEL-D, respectively [4]. MELs have high biodegradability, good stability at various conditions, and excellent emulsifying activity. Especially MEL-A has a low critical aggregate concentration (CMC) of 4.0×10^−6^ M with an excellent surface and interfacial tension-lowering activity [5]. As natural sugar-based surfactants, MEL-A can self-assemble into specific sponge, cubic, and lamella phases [6]. They also form giant vesicles or nanostructures that are used as models for cellular and molecular process studying [7]. These unique features expand its applications in the fields of nanomaterials, bioscience, macromolecular chemistry, cosmetics and medicines.

MELs show interesting biological activities. They can induce cell differentiation and apoptosis in human promyelocytic leukemia cell lines by decreasing the activity of the intracellular phospholipid and Ca^2+^ dependent protein kinase C [8], down-regulate the tyrosine kinase activities of K562 cells to induce the cell differentiation [9], and inhibit the secretion of inflammatory mediators from mast cells with the inhibition of Ca^2+^ increase and phosphorylation of MAP kinases [10]. Accordingly, they also possess antioxidant activity or repair damaged cells toward human skin [11]. In consideration of the various beneficial properties of MELs, a continuing effort to explore the potential values of MEL-A was carried out in the current study. *Pseudozyma aphidis* has been reported to be a useful strain for the production of MELs from soybean oil [12]. Therefore, MELs were derived from *Pseudozyma aphidis* under optimized conditions obtained from our previous studies, and the structural and surface parameters of the major MEL-A type were identified. Additionally, the effects of them on B16 melanoma cells, including the induction of differentiation and apoptosis, as well as the possible signaling pathway were evaluated deeply.

## Materials and Methods

### Biosynthesis of MELs

*Pseudozyma aphidis* DSM70725 was purchased from Deutsche Stammsammlung für Mikroorganismen und Zellkulturen, Braunschweig, Germany. This fungus produces no endotoxin. Stock cultures (3.0 g yeast extract, 3.0 g malt extract, 5.0 g peptone, 10.0 g glucose, 15.0 g agar, and 1.0 L distilled water) were grown at 28°C for 4 days. The yeast cells were inoculated to 250 mL culture flasks which contained glucose 40.0 g/L, yeast extract 1.0 g/L, NaNO_3_ 3.0 g/L, MgSO_4_·7H_2_O 0.3 g/L, and KH_2_PO_4_ 0.3 g/L (pH 6.0), and incubated at 28°C on a 180 rpm rotary shaker for 2 days. When finished, 10 mL/L seed culture was transferred to the basal liquid culture medium and then incubated on a 180 rpm rotary shaker at 28°C for 7 days for MEL biosynthesis. This process was carried out in the optimized basal liquid cultures which contained soybean oil 96.8 mL/L, yeast extract 1.5 g/L, peptone 1.0 g/L, NaNO_3_ 1.5 g/L, MgSO_4_·7H_2_O 0.6 g/L, MnSO_4_ 0.1 g/L, CaCl_2_ 0.03 g/L.

### Extraction and identification of MELs

After the fermentation, 50.0 mL of culture suspension was vigorously mixed with 50.0 mL ethyl acetate and centrifuged for 10 min at 4,000 rpm. The supernatant was extracted and evaporated. This procedure was repeated with the sediment for two times. Next, the transparent and sticky MELs were extracted twice with cyclohexane and methanol to remove the remaining oil and fatty acids. The crude MELs was isolated and purified though silica gel column according to the method of Onghena et al [13], followed by analyzing with TLC (Silica gel 60 F, chloroform: methanol: water = 70:15:2, v/v), liquid chromatography-mass spectrometry (LC-MS), gas chromatography-mass spectrometry (GC-MS) and nuclear magnetic resonance (NMR) analysis.

For LC-MS, Agilent-1200 HPLC system (Agilent Technology, USA) was connected with MS spectrometer (LCD Deca xp max, Thermo Electron Corporation). A 5 μm (250 mm × 4.6 mm) Agilent ZORBAX SB-C18 column was used. Mobile phase consisted of solvent A (distilled water with 0.1% formic acid) and solvent B (acentonitrile). The elution was conducted at a flow rate of 0.2 mL/min in a linear gradient ascending: solvent B started from 50% to 65% within 10 min, then increased to 80% within 40 min, to 90% within 10 min, sustaining for 10 min at last. The ionization parameters were adapted to the flow rate and the mass range (300–2000). A drying temperature of 325°C was applied together with a drying gas (N_2_) at a flow of 10 mL/min, a capillary voltage of 2,500 V, a corona voltage of 4,000 V, and a nebulizer pressure of 35 psi. The injection volume was 10 μL in every test. The GC-MS analysis was performed according to the method of Fan et al [14]. For the analysis of NMR, 10.0 mg MEL-A was dissolved in 0.5 mL CDCl_3_ (99.9%). Both ^1^H NMR and ^13^C NMR spectra were recorded at 25°C by a Bruker AVIII 600 M instrument with TMS as an internal standard.

### Determination of surface tension and size of MEL-A in the solution

The surface tension and critical micelle concentration (CMC) of the glycolipid was determined pendant-drop method at 20°C, which was performed using Contact Angle Analizer (OCA 20, DATAPHYSI, Germany). The sizes of MEL-A solution at different concentrations were tested though Laser nanometer size analyzer (Malvern, UK).

### Cell culture

Mouse B16 melanoma cells and NIH3T3 cells were purchased from Shanghai cell bank of China and maintained in RPMI-1640 (Hyclone) supplemented with 10% fetal bovine serum (Hyclone) and 1% solution of 10^5^ u/L penicillin and 100 mg/L streptomycin, at 37°C in a humidified atmosphere of 5% CO_2_. The B16 cells were passaged once every two days.

### Growth inhibition assay

The B16 cell growth inhibition was determined by MTT (3-(4, 5-dimethylthiazol-2-yl)-2, 5-diphenyltetrazolium bromide) assay. B16 cells (10^5^ cells /mL) were placed in 96-well plates for 24 h. Following treatment with 0–25.0 μg/mL of the purified MELs (referred to MEL-A), the proliferation activity of the cells was tested by adding 5.0 mg/mL MTT after 24 h and 48 h of incubation. The absorbance was measured at 570 nm using a Multiskan (Thermo Electron Corp, Asheville, NC). The cell viability was expressed as a percentage of the control culture value which was considered to be 100% viable.

### Flow cytometric analysis of apoptotic cells and cell cycles

The B16 cells on logarithmic phase were seeded on six-well plates for 24 h, treated with various concentrations (0–25.0 μg/mL) of MEL-A for 24 h and then were collected and washed twice with PBS without Mg^2+^ and Ca^2+^ ions. For apoptosis analysis, the cells were suspended by binding buffer, labeled with 5 μL Annexin V-FITC and 5 μL PI (Sigma) individually, staining at room temperature for 5 min in the dark, and then were performed using FC 500 MCL flow cytometer in an hour. In terms of cell cycle distribution analysis, the obtained cells were fixed in 70% ethanol overnight and were precipitated using centrifugation at 1000 rpm for 5 min at 4°C, followed by stained with 10.0 μg/mL of PI (Sigma) for 15 min at room temperature in the dark. The prepared cells were determined using FC 500 MCL flow cytometer.

### Real-time fluorescence quantitative PCR analysis

Approximately 1×10^7^ B16 cells which were treated with or without 15.0 μg/mL MEL-A for 24 h were lysed in Trizol Reagent (Takara, Japan). Total RNA of the cells was prepared according to the manufacturer’s instructions. The RNA integrity was assessed by an Agilent 2100 Bioanalyzer (Agilent Technologies, Palo Alto, CA). RT-PCR was examined by Nanodrop (Thermo Scientific, USA). First-strand cDNA synthesis was carried out on 2 μg of the total RNA from samples with the PrimeScript*RT Master Mix according to the manufacturer’s instructions (Takara, Japan). RT-PCR was conducted according to the SYBR Green method (Roche FS Universal SYBR Green Master, Switzerland). Forward and reverse primers were designed and the sequences were designed as follows: CHOP-F ‘CAGCGACAGAGCCAGAAT’, CHOP-R ‘CAAGGTGAAAGGCAGGGA’; GRP78-F ‘GAAGGAGGATGTGGGCACG’, GRP78-R ‘CGCATCGCCAATCAGACG’; Bcl-2-F ‘AGAGCGTCAACAGGGAGA’, Bcl-2-R ‘AGCCAGGAGAAATCAAACAG’; Caspase-12-F ‘TGGAAGGTAGGCAAGACT’, Caspase-12-R ‘ATAGTGGGCATCTGGGTC’; Caspase-3-F ‘CTAATCTGACGGTCCTCC’, Caspase-3-R ‘TCGCCAAATCTTGCTAAT’; GAPDH-F ‘ACCACAGTCCATGCCATCAC’, and GAPDH-R ‘TCCACCACCCTGTTGCTGTA’. RT-PCR included preincubation at 95°C for 10 min, followed by 40 cycles at 95°C for 5 s, 60°C for 30 s and 72°C for 30 s, and 72°C for 10 min by one cycle. The relative expression of mRNA was calculated using the 2^−ΔΔCt^ method [15].

### Western blotting analysis

After treatment with MEL-A for 24 h, approximately 1×10^7^ cells were washed with cold PBS buffer twice, harvested and disrupted in lysis buffer. The cell lysate was centrifuged at 14,000 rpm for 10 min at 4°C, and the protein concentration in the supernatant was determined with the Bradford protein assay kit (Takara, Japan). In short, equal amounts of protein were separated by sodium dodecyl sulfate-polyacrylamide gel electrophoresis (SDS-PAGE) and then electrotransferred onto nitrocellulose. The membrane was clogged for 1.5 h with 5% defatted milk in TBST buffer (10 mM Tris-HCl, 150 mM NaCl, 0.05% Tween-20). The membranes were incubated with corresponding primary antibodies (Cleaved caspase-3, Caspase-3, KG22205; Bcl-2, KGP22169; Caspase-12, CST2202P; CHOP, KGYT0911; GRP78, KG22444; GAPDH, KGAA002; IRE, AI601; ATF6, SC22799), washed three times with TBST, and incubated with secondary antibodies (IgG-HRP, KGAA35) against the corresponding primary antibodies. The signals were visualized utilizing Chemiluminescence Imaging (SYNGENE G: BOXChemiXR5). The signal intensities of proteins were analyzed by the software Gel-Pro32.

### Statistical analysis

All the treatment effects were analyzed using analysis of variance. Differences were considered to be significant at *P* < 0.05 throughout the present study. Each point was the average of replicate experiments.

## Results

### MEL-A was identified as the major type

**Fig 1:**
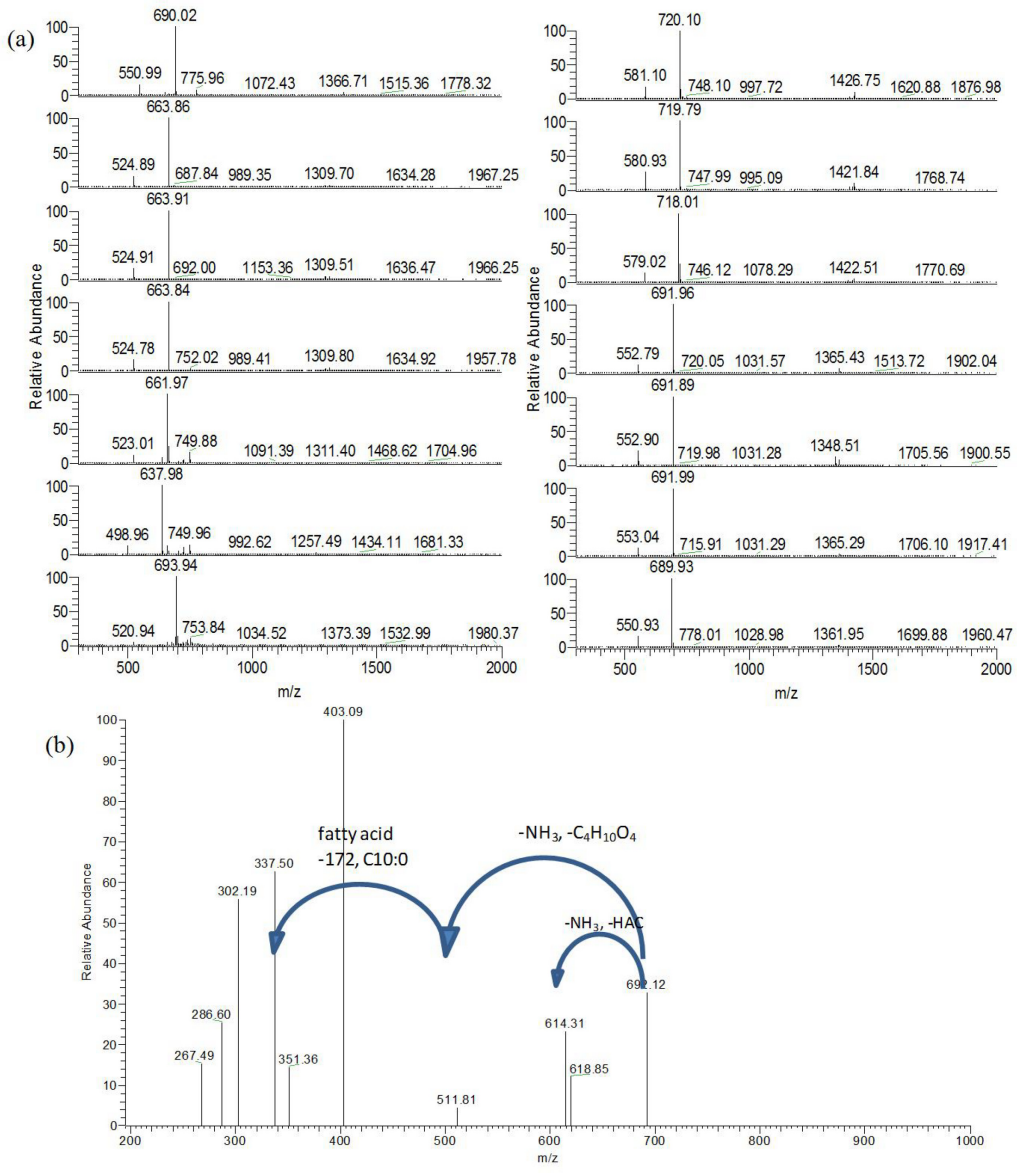
LC-MS results of MEL-A. (a), Mass spectra of different homologs isolated from the cultivation medium optimized by LC-MS. The units of CH_2_ or 2H molecular weight differences which are resulted from the different lengths of fat acid chain and the degree of unsaturation present in these compounds. (b), Loss of the erythritol, fatty acids and acetic acid was shown in the MS^2^ spectra.

**Fig 2:**
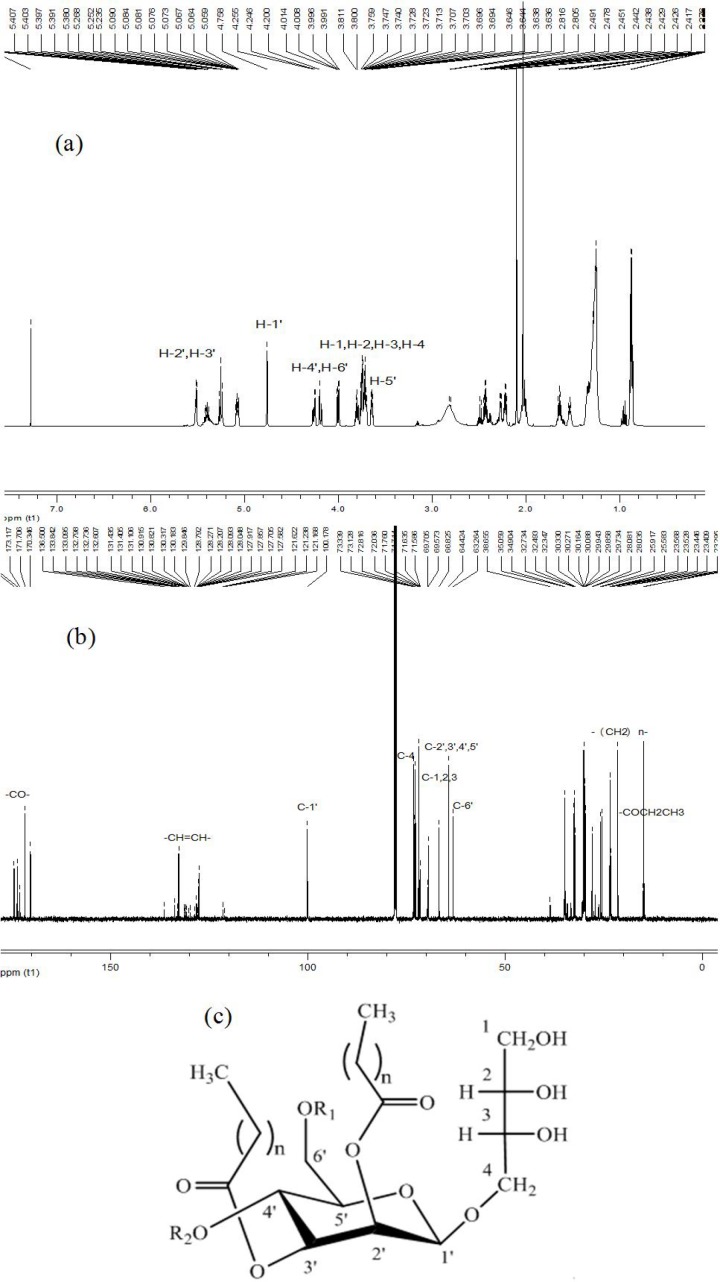
^1^H NMR spectrum and ^13^C NMR spectrum of MEL-A. (a), ^1^H signals at 0~7.0 ppm and (b), ^13^C signals at 0~180.0 ppm. (c), Structure of MELs, MEL-A: R_1_ = R_2_ = Ac; MEL-B: R_1_ = Ac, R_2_ = H; MEL-C: R_1_ = H, R_2_ = Ac; MEL-D: R_1_ = R_2_ = H, n = 6–16.

Crude MELs obtained from the liquid were further purified through silica gel column. The eluent fractions with the same *R*_*f*_ value on the TLC plate were collected for the next determinations. The first eluent with *R*_*f*_ value of 0.68 which was shown as MEL-A was detected by means of LC-MS. Derived from **Fig 1A**, we can see that the ion detected was m/z [M+NH_4_]^+^ with a mass-to-charge ratio of 636–720. Several isomers appeared in the closer retention time. Those homologs (shown in **Table 1**) had 2-unit mass differences and possessed different unsaturated bonds in the fatty acid moiety. Additionally, a characteristic pattern of mass spectrum was observed, and the ions corresponding to [M+NH_4_]^+^ had a 139 m/z difference from the simultaneously detected ions. According to the previous study [13], the ions with low mass was related to the loss of the erythritol moiety. The mass-mass spectrum of one of these compounds was shown in **Fig 1B**. The loss of the erythritol (-C_4_H_10_O_4_), fatty acids (C10:0, 172) and acetic acid (-HAc) was demonstrated. The structures of other homologs (M1-M9) were summarized in **Table 1**. Fatty acid profiles of MEL-A identified by GC-MS showed that the detected components were with a carbon length of C8 (18.22%), C10 (28.89%), C12 (13.11%), C14 (18.37%), C16 (6.17%), and C18 (7.25%). However, the corresponding MEL-A (**Table 1**) was deduced to possess C8, C10, C12 and C14 with zero, one or two double bonds in terms of the mass results and the structure of MELs (**Fig 2C**). It was possible that the fatty acids with C16 and C18 was not linked with MEL-A. We can also find that the fatty acids contained in the glycolipids were degraded by C2 units. The partial β-oxidation of fatty acids pathway was probably involved in the fermentation process [16]. Compared with MEL-A from the mutagenic strain *Pseudozyma aphidis* ZJUDM34 [14], these components which were incorporated much shorter fatty acid chains had a higher solubility. This property is useful for forming the microemulsion systems.

**Table 1:** Detected MEL-A homologs with various masses and fatty acids chain combinations.

NO.	[M+Na]^+^	[M+NH_4_]^+^	[M+NH_4_-C_4_H_10_O_4_]^+^	Molecular mass	Structure of fatty acids	Relative quantity (%)
M1	642.9	637.9	498.8	619.9	C8:0-C8:0	2.15
M2	666.9	661.9	522.9	643.9	C8:0-C10:2	6.43
M3	669.9	663.9	524.9	645.9	C8:0-C10:1 or C8:1-C10:0	22.89
M4	670.9	665.9	526.9	647.9	C8:0-C10:0	9.81
M5	695.0	690.0	550.7	672.0	C8:0-C12:2	19.2
M6	698.9	693.9	550.8	675.9	C8:0-C12:0	2.99
M7	696.7	691.7	552.9	673.7	C10:0-C10:1	19.65
M8	723.0	718.0	579.0	700.0	C10:1-C12:0 or C8:0-C14:2	1.56
M9	725.1	720.1	581.1	702.1	C8:1-C14:0	6.12

To further confirm the detailed structural type of MELs produced by this strain, NMR analysis was finally employed. As shown in **Fig 2A**, the resonances of ^1^H NMR δ 3.6–4.0 m were assigned to H1-H4 of the moiety of erythritol, and δ 4.7 d, δ 5.2 dd, δ 5.0 dd, δ 4.2–4.7 m, δ 3.6–3.7 m, and δ 4.2 m were referred to H1’-H6’ of the mannose, respectively. Chemical shifts of δ 1.2–1.4 m (-(CH_2_)n-), δ5.6–5.8 b (-CH = CH-), δ1.4–1.8 b (-OCOCH_2_), δ1.9–2.1 m (-CH_2_-CH = CH-), δ2.4–2.8 b (-OCOCH_2_-R_2_/R_3_) and δ 0.8 b (-CH_3_) were also detected by ^1^H NMR. **Fig 2B** showed that the key groups defined MEL types were signals at ^13^C NMR δ173.7–174.5 (-CO-) and δ 21.4 b (-COCH_2_CH_3_). Chemical shifts of δ 100.2 d (C-1’), δ 69.5 d (C-2’), δ 71.6 d (C-3’), δ 66.8 d (C-4’), δ 73.3 d (C-5’), δ 63.2 t (C-6’), δ 64.4 t (C-1), δ 72.8 d (C-2), δ 72.0 d (C-3), δ 73.1 t (C-4), δ 23.2–32.7 (-(CH_2_)n-), δ 121.6–136.5 (-CH = CH-), and δ 15.1–16.2 (-CH_3_) were detected corresponding to the groups of MEL structure. In consideration of the above LC-MS and GC-MS data, the type of MELs identified as MEL-A with short and medium fatty acids was confirmed. Generally, a mixture compound was adopted for activity investigations. As a result, we used the purified MEL-A with shorter fatty acid chains (C8-C14) to examine the biological activity of this biosynthetic glycolipid.

### Physicochemical properties of MEL-A

**Fig 3:**
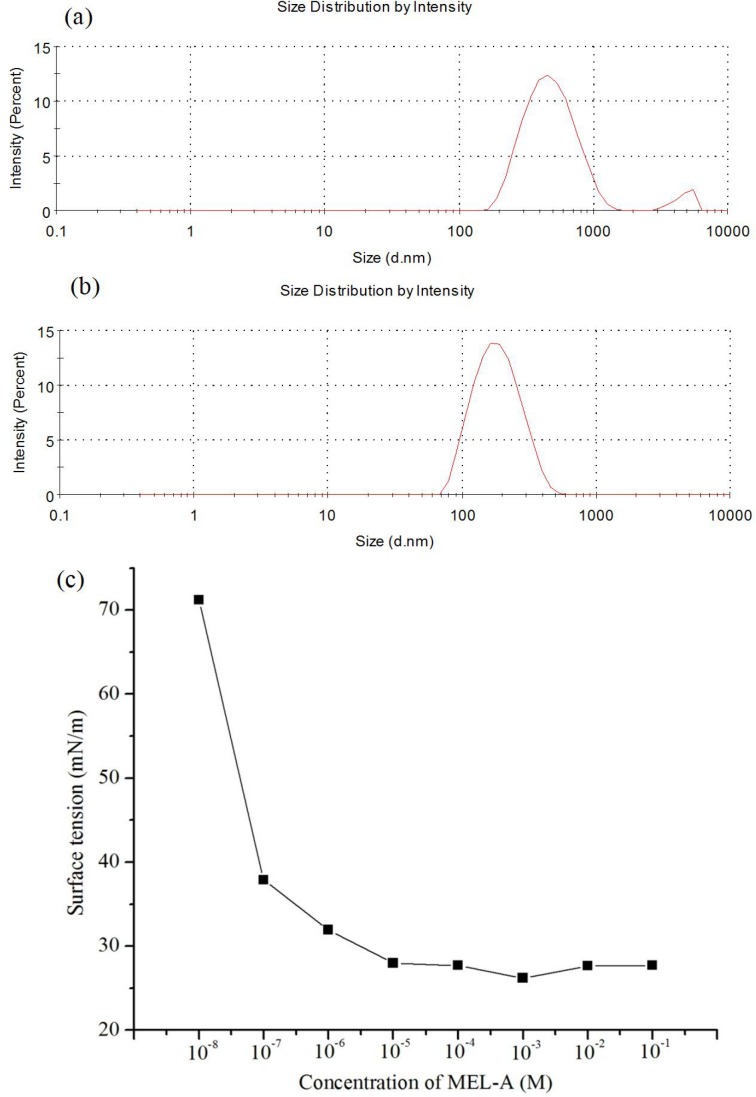
The solution state of MEL-A in water. (a), mean particle sizes of MEL-A solution at the concentrations of 12.0 mg/L. (b), mean particle sizes of MEL-A solution at the concentrations of 12.0 μg/L. (c), the surface tension of MEL-A changed with various concentrations. The concentration at the curve break records the critical micelle concentration (CMC).

Characterizing the surface properties of biosurfactants is crucial for of their special applications. One of the most important properties of surfactants is to form micelles in aqueous solution. We thus further investigated the surface-active properties of MEL-A, which showed that the CMC of MEL-A was 15.0 mg/L (about 10^−5^ M), and the corresponding surface tension was 27.69 mN/m (**Fig 3C**). Moreover, MEL-A exhibited an excellent surface-tension lowering activity by decreasing the lipid surface tension to 31.14 mN/m at this CMC value. The sizes of MEL-A solution at the concentration of 12.0 mg/L were approximately 450±2.05 nm and at 12.0 μg/L were 141±1.22 nm (as shown in **Fig 3A and 3B**). In general, MEL-A self-assembles into nanostructures and thermodynamically stable vesicles over 1 mM [7]. MEL-A can interact with biomolecules of cells when applied to drug delivery or gene transfections. This property may improve its effects on cells.

### MEL-A induces growth inhibition of B16 cells

**Fig 4:**
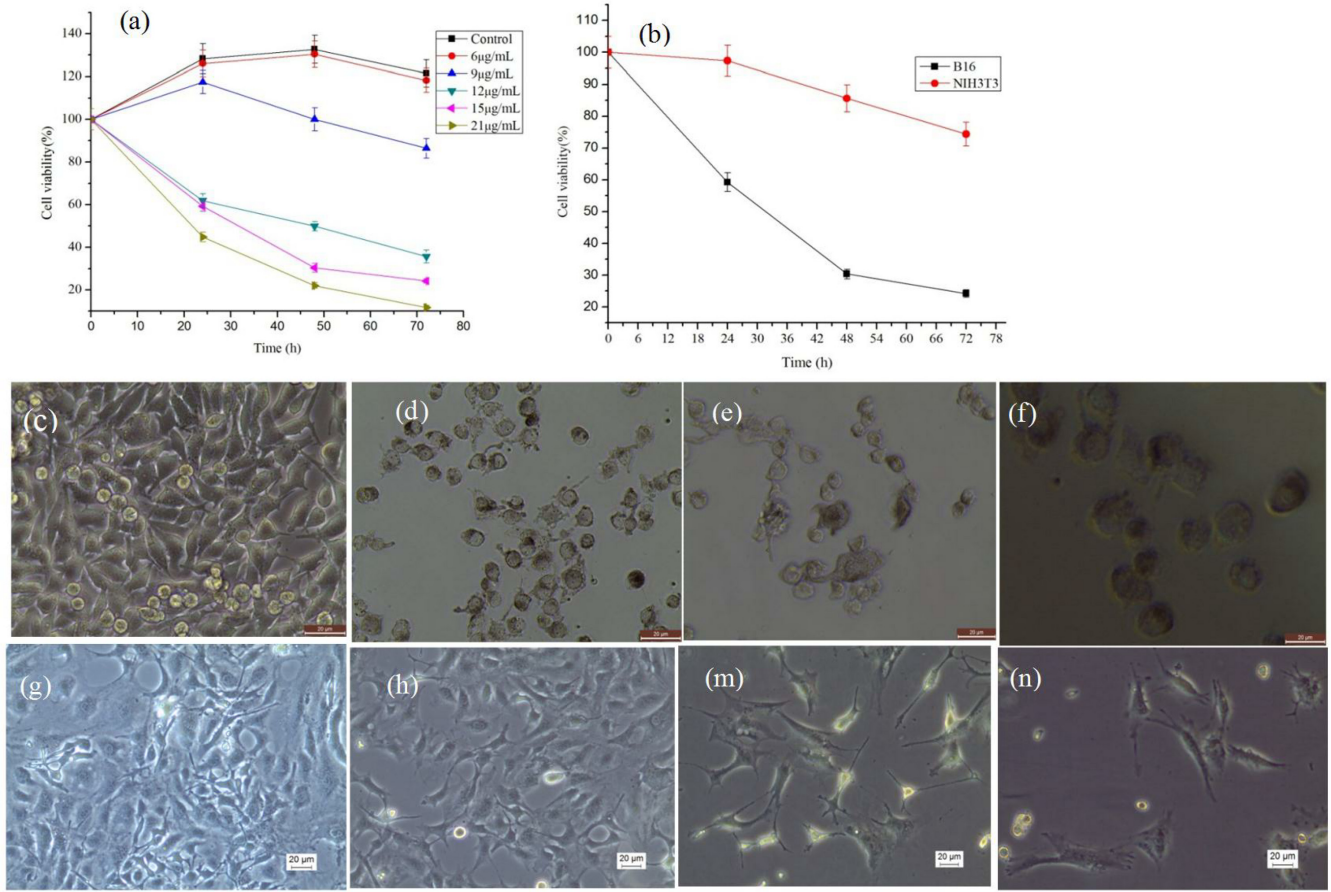
The viabilities of B16 cells and NIH3T3 cells in response to MEL-A treatments. (a), B16 cells treated with increasing doses of MEL-A had obvious changes. (b), the viability of the normal NIH3T3 cells treated by 15.0 μg/mL MEL-A for 72 h. (c, d, e, f) refers to the photographs of untreated B16 cells, and cells treated with MEL-A for 24 h, 48 h and 72 h. (g, h, m, n) refers to the photographs of untreated NIH3T3 cells and cells treated with MEL-A for 24 h, 48 h, and 72 h, respectively.

In the preliminary experiments, we have conducted the comparison of the cytostatic effects of MEL-A against three melanoma cell lines, B16-4A5, B16-F0 and B16 cells, and at last the B16 cells was chose for the further testing. As control, the normal NIH3T3 cells were used for cytotoxicity test. The effects of MEL-A solution on the proliferation and viability of B16 cells and NIH3T3 cells were determined by means of MTT assay. In **Fig 4A**, MEL-A exhibited a strong inhibition against B16 cell growth in a time-dependent and dose-dependent manner. But the viability of NIH3T3 cells was higher than that of B16 cells at the same concentration of MEL-A solution (shown in **Fig 4B**). The NIH3T3 cell population was decreased probably due to the nutrition starvation over time. **Fig 4G, 4H, 4M and 4N** showed the morphology of NIH3T3 cells treated with MEL-A. No obvious apoptosis changes were observed. And the cells with irregular shape could result from the cell states. However, obvious morphology variations in B16 cells treated with 12.0 μg/mL MEL-A were found in **Fig 4C, 4D, 4E and 4F**. In contrast to the fibroblast-like control cells, the cells treated by MEL-A were out of shape with irregular cell membrane and condensed cytoplasm. This is a significant characteristic of cell apoptosis. The melanin was also observed to secret into the cell culture liquid. It could be attributed to the changes of the cell cytomembrane permeability in the presence of this kind of glycolipid biosurfactant.

### Apoptosis and cell cycle distribution of MEL-A-induced B16 cells

**Fig 5:**
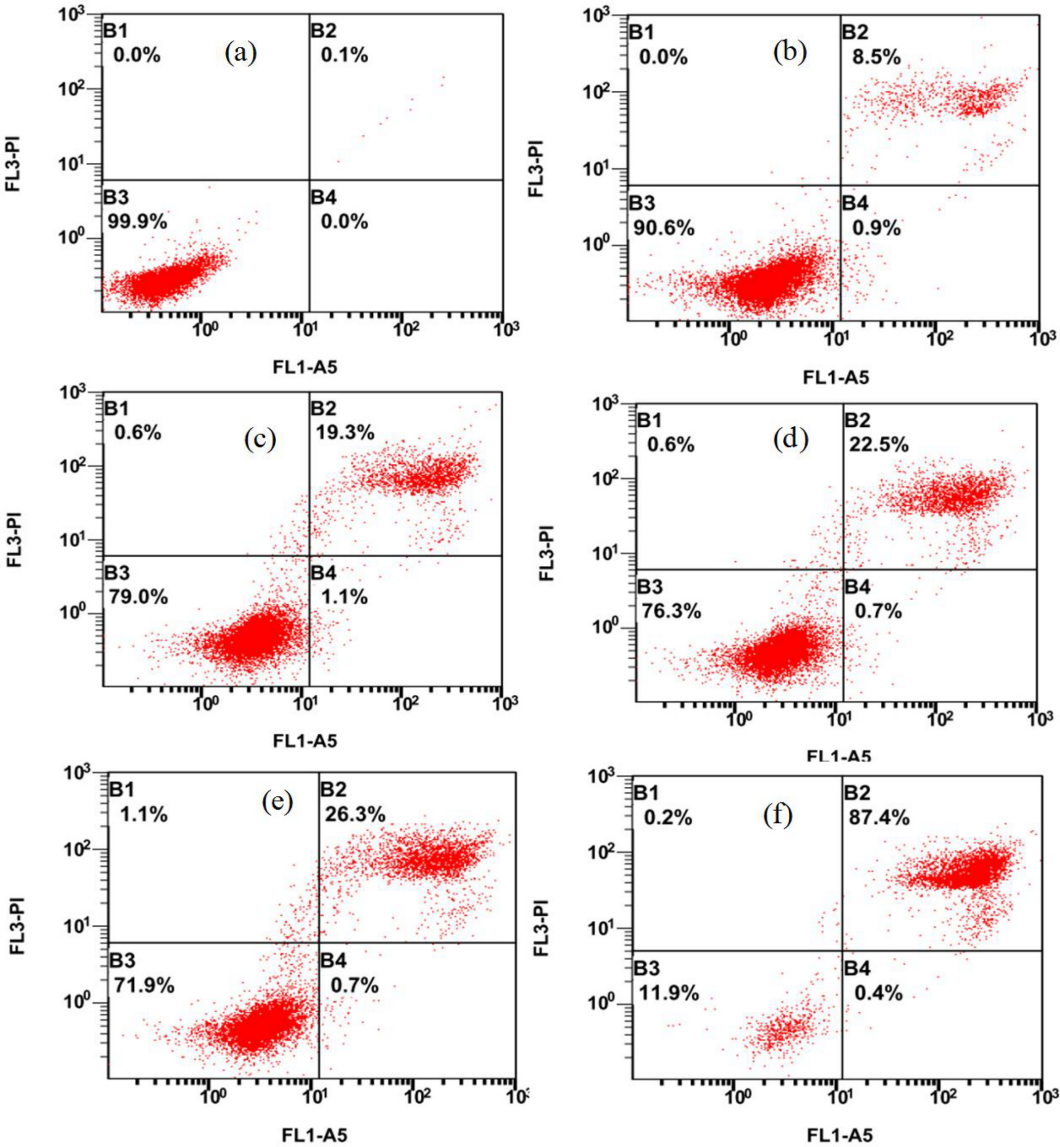
Effects of MEL-A on apoptosis of B16 cells. Cells were treated with different concentrations of MEL-A for 24 h. (a) ~ (f) refer to the cell population changes of B16 treated by 0, 6.0, 9.0, 12.0, 15.0, 25.0 μg/mL MEL-A, respectively. Most MEL-A-induced cells were evident in B2 fraction, and the tendency of the induced cells apoptosis was in a dose-dependent manner. Mean ± SD, n = 3.

To further confirm and quantify the apoptosis effect of B16 cells triggered by MEL-A, cells treated with MEL-A for 24 h were stained with both Annexin V-FITC and PI, and subsequently analyzed by flow cytometry. **Fig 5** demonstrated that early apoptotic cells appeared in the Annexin V+/PI-fraction (B4), and late apoptosis or necrotic cells were present in the Annexin V+/PI-fraction (B2). Most MEL-A-induced cells were evident in B2 fraction. In addition, the corresponding quantities of necrosis and apoptosis increased with the increasing MEL-A concentrations. This finding further confirmed that MEL-A induced cells apoptosis was in a dose-dependent manner.

**Fig 6:**
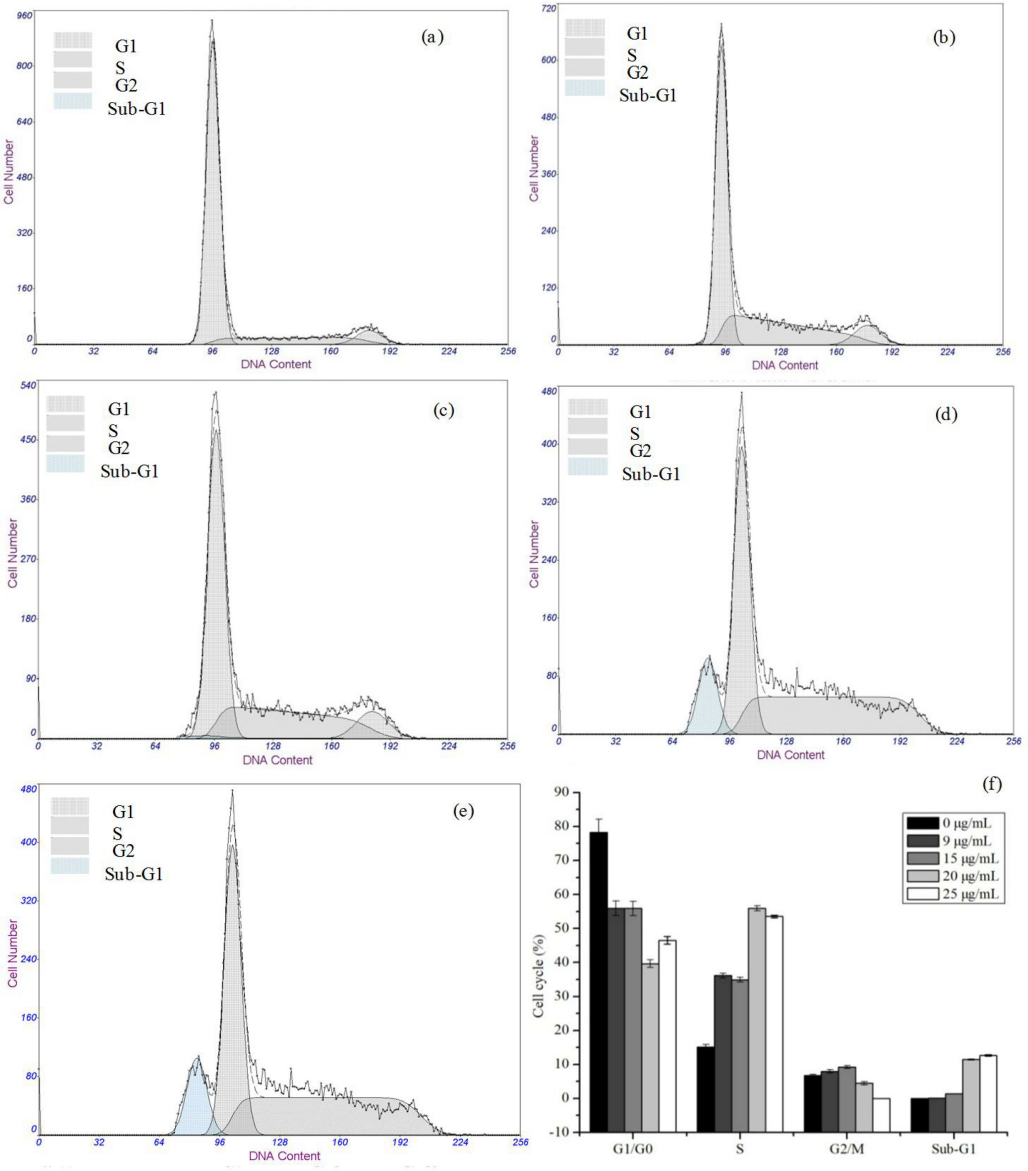
Effects of MEL-A on cell cycle distributions of B16 cells. Cells were treated with different concentrations of MEL-A for 24 h. (a) ~ (e) refer to the cell cycle distributions of B16 cells treated by 0, 9.0, 15.0, 20.0, 25.0 μg/mL MEL-A, respectively. G1/G0, S, G2/M and Sub-G1 indicate the different cell phases. Mean ± SD, n = 3. The results in (f) summarized the relative ratios of each cell cycle, indicating that MEL-A caused cell cycle arrest at the S phase. The cell population of Sub-G1 phase was slightly increased with exposure to above 15.0 μg/mL of MEL-A.

Next, cell cycle distribution as one of apoptotic characteristics was investigated by flow cytometric analysis. As shown in **Fig 6**, MEL-A induced a remarkable increasing S-phase arrest of B16 cells in contrast to the control. The percentage values of cells exposed to 0–25.0 μg/mL of MEL-A in S phase were as follows: 15.09%, 36.13%, 34.90%, 55.91%, and 53.52%. The increase of cell population at S phase was accompanied by a decrease in the G1/G0 and G2/M phase. Meanwhile, the cell population of Sub-G1 phase was slightly increased with exposure to above 15.0 μg/mL of MEL-A. These results indicated that MEL-A caused cell cycle arrest at the S phase, followed by the cell apoptosis. The present finding was different from the previous study that MEL induced cell cycle arrest at the G1/G0 phase of the cycle [17]. The effective dose of MEL-A, the various cell states and homeostasis may contribute to this difference. Consequently, the signaling pathways of the different dose-inducing apoptosis need to be elucidated.

### Endoplasmic reticulum stress pathway was involved in MEL-A-induced cell apoptosis

**Fig 7:**
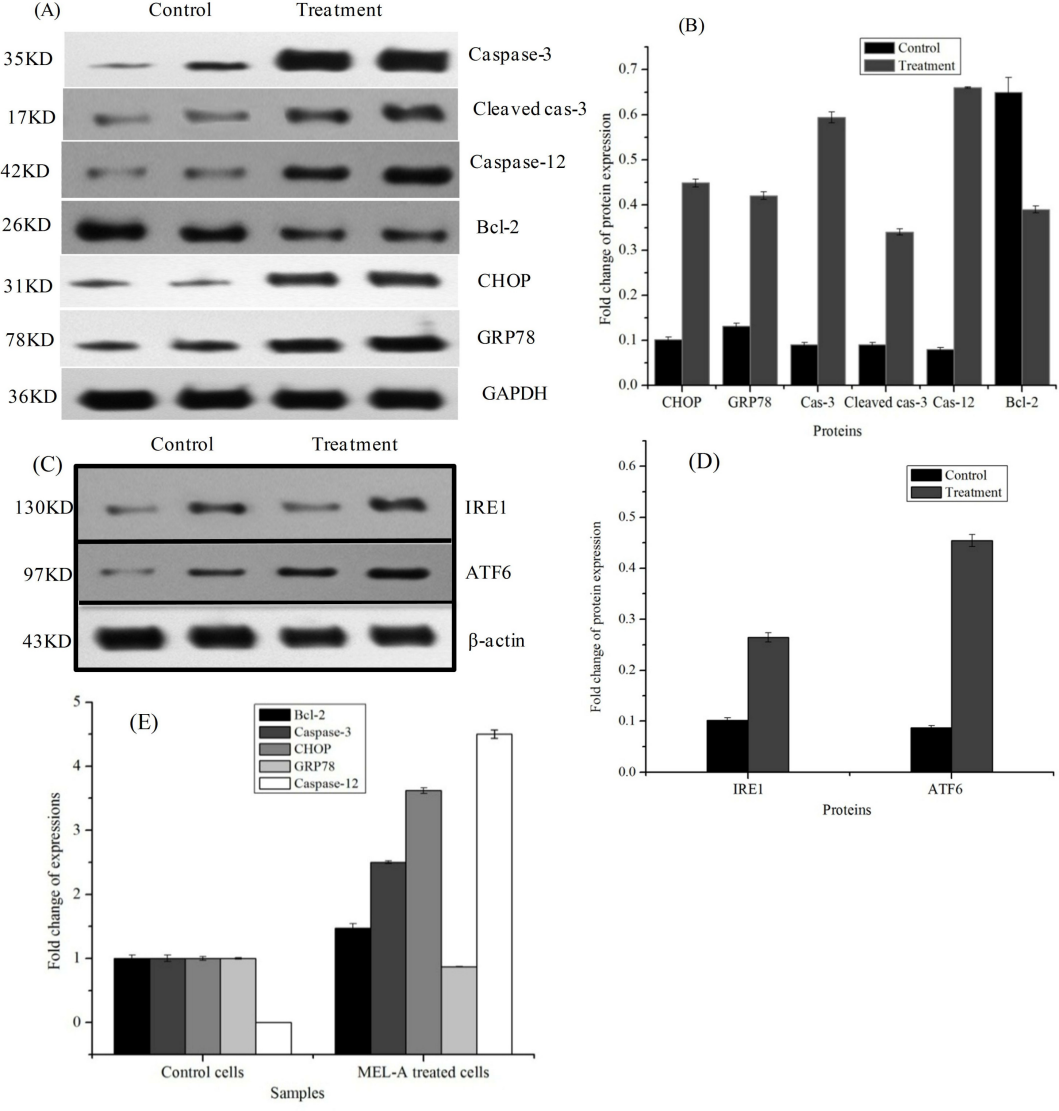
Effects of MEL-A on the protein expression and mRNA expression in B16 cells. (A) and (B), protein expression analysis of Caspase-3, cleaved Caspase-3, Caspase-12, Bcl-2, CHOP and GRP78 in the B16 cells treated with 15.0 μg/mL MEL-A for 24 h. The untreated cells were used as control. (C) and (D), fold changes of IRE1 and ATF6 protein expression. (E), mRNA expression analysis of Bcl-2, Caspase-3, CHOP, GRP78 and Caspase-12 in the B16 cells treated with 15.0 μg/mL MEL-A for 24 h. The untreated cells were used as control. Mean ± SD, n = 3.

In this work, we made efforts to explore the mechanism of MEL-A induced melanoma cell apoptosis. To determine the possible targeted pathway of B16 cells apoptosis triggered by MEL-A, the mRNA expressions of Bcl-2, Caspase-12, Caspase-3, CHOP and GRP78 were analyzed by qRT-PCR, and the protein expressions of Caspase-3, CHOP and GRP78 were further measured by western blotting analysis. As shown in **Fig 7E**, mRNA expressions of Bcl-2, Caspase-3, CHOP and GRP78 increased by 1.47, 2.50, 3.62 and 0.87 times, respectively. Caspase-12 was also proved to be an important signal factor in endoplasmic reticulum stress (ERS) signaling pathway [18]. No evidence showed Caspase-12 expressed in the control cells, but its expression was obvious in those MEL-A-treated cells.

We further used immunoblotting to monitor the changes of key factors associated with ERS. Western blotting results presented in **Fig 7A** and **7B** showed that Caspase-12, cleaved caspased-3, Caspase-3, CHOP, and GRP78 in cells revealed an increasing tendency of protein expressions after the treatment of MEL-A. The relative expression increased by 7.25, 2.78, 5.16, 3.41, and 2.38 times, respectively. In **Fig 7C** and **7D**, it revealed an upregulation trend of protein IRE1 and ATF6 with fold changes of 1.68 and 4.70 times. However, the expression of protein Bcl-2 decreased by 1.28 times.

## Discussion

MEL-A was reported to be the major product from soybean oil by *P*.*aphidis* DSM70725. It represented the diacetylated compound which was acetylated at C-4’ and C-6’ position in the carbohydrate backbone, mannopyranosyl. The fatty acids were contained as the hydrophobic groups. Because of the different carbon length and unsaturation degree of fatty acids, it is still difficult to isolate each kind of MEL-A homologs [19]. And much more costs are needed to obtain the monomer. Thus, in this work, we measured the relative content of each homolog and applied the mixed MEL-A homologs for the activity tests. We predicted that the combined action were higher than that of a single function. To confirm this, the monomer is needed. More strategies, such as developing efficient and cheap product recovery of downstream processing and novel genetically engineered strains, are in progress.

In recent years, the development of the nanotechnology area was accelerated by the new comings of functional structures which are formed though self-assembling properties of amphiphilic molecules [7]. As a natural biological amphiphilic molecule, MEL-A can self-assemble into the ordered structures of sponge, coacervates, vesicles at various concentrations by using hydrogen bonding, hydrophobic and van der Waals interactions. It interacted with biomolecules of cells when was applied to drug delivery vehicles and gene transfections. What’s more, MEL-A may show binding affinity towards the membrane proteins and phospholipids because of its self-assembling properties. We thus proposed that the interaction of MEL-A with cells might be related to some proteins or phospholipids in the cell membrane.

On the other hand, MEL-A produced from different substrates displayed different interfacial tensions. Its excellent surface/ interfacial tension-lowering activity could contribute to the cell apoptosis induced by MEL-A. Because the permeability of cell membrane was likely to change in exist of surfactants.

For cytotoxicity examinations, three cells of B16-4A5, B16-F0 and B16 cells were used. MEL-A had no cytotoxicity to these three cell types at the test concentrations. All the B16 cells were bigger and have obvious morphology. B16-4A5 and B16-F10 were obtained by selective procedures. In view of the passage stability and cell viabilities of these cells, B16 cells were chosen for further experiments. Normal NIH3T3 cells were determined as control. The data revealed MEL-A had no cytotoxicity to the normal cells. Therefore, we considered that MEL-A was a suitable candidate used for the following experiments.

The pharmacological activity of MELs has been studied a lot in the past decades. The mechanisms are still need to be established. MELs were reported to induce P12 cells apoptosis and differentiation through an ERK-related signal cascade and trigger the differentiation of B16 melanoma cells via a signaling pathway that involves PKCα [20, 21]. Recently, endoplasmic reticulum stress was proved to be a major approach for anti-melanoma therapy. It can result in melanoma cells apoptosis by inducing abnormal protein glycosylation and autophagy [22]. As seen in Fig 4D, cell vacuolization increased after treatment with MEL-A. Combined with the results of the unfolded proteins, autophagy process which is a self-defensive reaction of the cells in response to glycolipids may also be activated. It may be a new mechanism of inducing the programmed B16 cell death other than cell apoptosis. The increasing evidences associated autophagy with an ERS [23, 24]. And the ERS was as well reported to be connected to autophagy by unfolded protein response (UPR) and calcium [25]. In MEL-A induced B16 cells, the changes of UPR signalings such as IRE1 and ATF6 were confirmed by western blotting. In the presence of ER stress, such proteins were cleaved from ER membrane on activation of the unfolded protein response. PERK (the PKR-like endoplasmic reticulum kinase), one of the ER transmembrane proteins, was also activated by the ER stress [26]. These signaling proteins activated the UPR, and the active UPR could protect cell damage caused by ER stress, but the excessive ER stress could also disable ER, which subsequently resulted in an apoptosis.

The proteins such as C/EBP homologous protein (CHOP), glucose-regulatory protein-78 (GRP78), Bcl-2 and caspases have long been recognized as key factors associated with ERS. The western blotting results revealed that the expression of CHOP increased and that of Bcl-2 had a down-regulation tendency. We then deduced that ERS contributed to the B16 cell apoptosis. As acknowledged, CHOP/GADD153 is the specific factors in ERS pathway, and its over-expression can lead to a down-regulation of Bcl-2 expression which triggers cell cycle arrest cell cycle arrest or cell apoptosis [27, 28]. Namely, Bcl-2 which is responsible for the cell apoptosis and cell death can antagonize apoptosis induced by protein CHOP/GADD153 and stimulation of ERS[29]. On the other hand, Bcl-2 was observed to regulate a common cell apoptosis pathway and functioned at various signal sites. The main action site of Bcl-2-related proteins is thought to be the mitochondrion [30, 31]. In fact, the Bcl-2 protein not only exists in mitochondrial membranes but also is present in ER membranes [32]. However, the regulatory effects of CHOP/GADD153 on MEL-A induced cell apoptosis need to be confirmed by using specific shRNA against CHOP to validate the deductions with employing a cell model system.

The expression of chaperon in GRP78 and the increase of Ca^2+^ concentration in the cytoplasm can trigger caspase activation, followed by cell apoptosis [33, 34]. In the MEL-A-induced cell apoptosis process, GRP78 proteins were expressed in order to prevent the unfolded and misfolded proteins which were accumulated by the changes of internal cell environment from the ER stress, and thus protect cells from apoptosis. Moreover, GRP78 could particularly trigger the activation of Caspase-12, followed by a continuing activation of caspases [35]. We found that the expression of Caspase-12 in B16 cells was greatly increased after treatment by MEL-A. These findings further confirmed the presence of ERS pathway. In addition, we found the expression of Cyto.c increased in the treated cells. It was quite possible that the release of Cyto.c and caspase activations could be resulted from the excessive ER stress [32, 36]. To elucidate this, we further determined the Cyto.c in the cytosolic fraction with Cytochrome c apoptosis assay kit (Biovision). However, the western blotting results revealed that no obvious stripes were increased. In this case, we speculated that the glycolipid did not cause the mitochondrial apoptosis pathway or the ER stress had no effect on the release of Cyto.c into the cytoplasm. Further validations are necessary to confirm this in our future work.

In conclusion, the bio-glycolipid incorporated medium and short fatty acid chains with a length of C8 to C14 and with no, one or two double bonds was identified as new homologs of MELs, MEL-A. Our results confirmed its cytostatic activity of triggering apoptosis of B16 cells in vitro. As the results mentioned above, MEL-A possibly acts on the cells by self-assembling into cytomembranes, interacts with the extracellular protein or phospholipid signal transducers, and induces the accumulation of unfolded proteins which can stimulate the activation of procaspase-12 and inductions of CHOP and GRP78. The simultaneous down-regulation of Bcl-2 expression is likely to trigger ER stress, and then induce cell apoptosis or death. We thus proposed that MEL-A induced the B16 cells apoptosis via the ER stress pathway, combined with the down-regulation of Bcl-2 and the up-regulation of caspase-12, caspase-3, cleaved caspase-3, CHOP and GRP78, as well as the UPR signaling proteins. More studies are still needed to focus on exploring uncharacterized mechanisms, as well as on accumulating more evidences of the relationship between the glycolipids and the cell-membrane receptors. That whether autophagy was active by the glycolipids deserves to be confirmed. It is anticipating that the applications of MEL-A in the fields of functional foods, cosmetics, environment and biotechnology are enlarged.

## References

[1] Arutchelvi JI, Bhaduri S, Uppara PV, Doble M. Mannosylerythritol lipids: a review. J Ind Microbiol Biot. 2008;35(12):1559–70. 10.1007/s10295-008-0460-4 . 18716809

[2] Dubey KV, Charde PN, Meshram SU, Shendre LP, Dubey VS, Juwarkar AA. Surface-active potential of biosurfactants produced in curd whey by *Pseudomonas aeruginosa* strain-PP2 and *Kocuria turfanesis* strain-J at extreme environmental conditions. Bioresour Technol. 2012;126:368–74. 10.1016/j.biortech.2012.05.024 . 22683199

[3] Kitamoto D. Extracellular accumulation of mannosylerythritol lipids by a strain of *Candida-antarctica*. Agr Biol Chem Tokyo. 1990;54(1):31–6. 10.1271/bbb1961.54.31

[4] Kim HS, Yoon BD, Choung DH, Oh HM, Katsuragi T, Tani Y. Characterization of a biosurfactant, mannosylerythritol lipid produced from *Candida* sp SY16. Appl Microbiol Biot. 1999;52(5):713–21. 10.1007/s002530051583 . 10570818

[5] Imura T, Ohta N, Inoue K, Yagi N, Negishi H, Yanagishita H, et al Naturally engineered glycolipid biosurfactants leading to distinctive self-assembled structures. Chem-Eur J. 2006;12(9):2434–40. 10.1002/chem.200501199 . 16374891

[6] Imura T, Hikosaka Y, Worakitkanchanakul W, Sakai H, Abe M, Konishi M, et al Aqueous-phase behavior of natural glycolipid biosurfactant mannosylerythritol lipid A: Sponge, cubic, and lamellar phases. Langmuir. 2007;23(4):1659–63. 10.1021/la0620814 . 17279642

[7] Dai K, Tomotake M, Tokuma F, Masa-aki K, Tomohiro I. Self-assembling properties of glycolipid biosurfactants and their potential applications. Curr Opin Colloid Interface Sci. 2009;14(5):315–28. 10.1016/j.cocis.2009.05.009

[8] Isoda H, Shinmoto H, Kitamoto D, Matsumura M, Nakahara T. Differentiation of human promyelocytic leukemia cell line HL60 by microbial extracellular glycolipids. Lipids. 1997;32(3):263–71. 10.1007/s11745-997-0033-0 . 9076663

[9] Isoda H, Nakahara T. Mannosylerythritol lipid induces granulocytic differentiation and inhibits the tyrosine phosphorylation of human myelogenous leukemia cell line K562. Cytotechnology. 1997;25(1–3):191–5. 10.1023/a:1007982909932 . 22358891 PMC3466747

[10] Morita Y, Tadokoro S, Sasai M, Kitamoto D, Hirashima N. Biosurfactant mannosyl-erythritol lipid inhibits secretion of inflammatory mediators from RBL-2H3 cells. Biochim Biophys Acta-Gen Subj. 2011;1810(12):1302–8. 10.1016/j.bbagen.2011.07.002 . 21777658

[11] Takahashi M, Morita T, Fukuoka T, Kitamoto D, Takahashi M, Tomohiro, et al Glycolipid biosurfactants, mannosylerythritol lipids, show antioxidant and protective effects against H_2_O_2_-induced oxidative stress in cultured human skin fibroblasts. J Oleo Sci. 2012;61(8):457–64. 10.5650/jos.61.457 . 22864517

[12] Rau U, Nguyen LA, Schulz S, Wray V, Nimtz M, Roeper H, et al Formation and analysis of mannosylerythritol lipids secreted by *Pseudozyma aphidis*. Appl Microbiol Biot. 2005;66(5):551–9. 10.1007/s00253-004-1672-9 . 15248042

[13] Onghena M, Geens T, Goossens E, Wijnants M, Pico Y, Neels H, et al Analytical characterization of mannosylerythritol lipid biosurfactants produced by biosynthesis based on feedstock sources from the agrofood industry. Anal Bioanal Chem. 2011;400(5):1263–75. 10.1007/s00216-011-4741-9 . 21318245

[14] Fan LL, Dong YC, Fan YF, Zhang J, Chen QH. Production and identification of mannosylerythritol lipid-A homologs from the ustilaginomycetous yeast *Pseudozyma aphidis* ZJUDM34. Carbohydr Res. 2014;392:1–6. 10.1016/j.carres.2014.04.013 . 24814655

[15] Livak KJ, Schmittgen TD. Analysis of relative gene expression data using real-time quantitative PCR and the 2(-Delta Delta C(T)) Method. Methods. 2001;25(4):402–8. 10.1006/meth.2001.1262 . 11846609

[16] Kitamoto D, Yanagishita H, Haraya K, Kitamoto HK. Contribution of a chain-shortening pathway to the biosynthesis of the fatty acids of mannosylerythritol lipid (biosurfactant) in the yeast *Candida antarctica*. Biotechnol Lett. 1998;20(9):813–8. 10.1023/A:1005347022247

[17] Zhao XX, Wakamatsu Y, Shibahara M, Nomura N, Geltinger C, Nakahara T, et al Mannosylerythritol lipid is a potent inducer of apoptosis and differentiation of mouse melanoma cells in culture. Cancer Res. 1999;59(2):482–6. . 9927066

[18] Nakagawa T, Zhu H, Morishima N, Li E, Xu J, Yankner BA, et al Caspase-12 mediates endoplasmic-reticulum-specific apoptosis and cytotoxicity by amyloid-beta. Nature. 2000;403(6765):98–103. 10.1038/47513 . 10638761

[19] Yu M, Liu Z, Zeng G, Zhong H, Liu Y, Jiang Y, et al Characteristics of mannosylerythritol lipids and their environmental potential. Carbohydr Res. 2015;407:63–72. 10.1016/j.carres.2014.12.012 . 25723622

[20] Zhao XX, Murata T, Ohno S, Day N, Song J, Nomura N, et al Protein kinase C alpha plays a critical role in mannosylerythritol lipid-induced differentiation of melanoma B16 cells. J Biol Chem. 2001;276(43):39903–10. 10.1074/jbc.M010281200 . 11546757

[21] Wakamatsu Y, Zhao XX, Jin CY, Day N, Shibahara M, Nomura N, et al Mannosylerythritol lipid induces characteristics of neuronal differentiation in PC12 cells through an ERK-related signal cascade. Eur J Biochem. 2001;268(2):374–83. 10.1046/j.1432-1327.2001.01887.x . 11168372

[22] Wang HM, Chen CY, Wu PF. Isophilippinolide A arrests cell cycle progression and induces apoptosis for anticancer inhibitory agents in human melanoma cells. J Agric Food Chem. 2014;62(5):1057–65. 10.1021/jf403730z . 24359513

[23] Fouillet A, Levet C, Virgone A, Robin M, Dourlen P, Rieusset J, et al ER stress inhibits neuronal death by promoting autophagy. Autophagy. 2012;8(6):915–26. 10.4161/auto.19716 . 22660271 PMC3427257

[24] Rashid HO, Yadav RK, Kim HR, Chae HJ. ER stress: Autophagy induction, inhibition, and selection. Autophagy. 2015:0. 10.1080/15548627.2015.1091141 . 26389781 PMC4824587

[25] Hoyer-Hansen M, Jaattela M. Connecting endoplasmic reticulum stress to autophagy by unfolded protein response and calcium. Cell Death Differ. 2007;14(9):1576–82. 10.1038/sj.cdd.4402200 . 17612585

[26] Patil C, Walter P. Intracellular signaling from the endoplasmic reticulum to the nucleus: the unfolded protein response in yeast and mammals. Curr Opin Cell Biol. 2001;13(3):349–56. 10.1016/S0955-0674(00)00219-2 . 11343907

[27] Oyadomari S, Mori M. Roles of CHOP/GADD153 in endoplasmic reticulum stress. Cell Death Differ. 2004;11(4):381–9. 10.1038/sj.cdd.4401373 . 14685163

[28] Sidrauski Carmela, Chapman Rowan, Walter aP. The unfolded protein response: an intracellular signalling pathway with many surprising features. Trends Cell Biol. 1998;8:245–9. 10.1016/S0962-8924(98)01267-7 . 9695849

[29] McCullough KD, Martindale JL, Klotz LO, Aw TY, Holbrook NJ. Gadd153 sensitizes cells to endoplasmic reticulum stress by down-regulating Bcl2 and perturbing the cellular redox state. Mol Cell Biol. 2001;21(4):1249–59. 10.1128/MCB.21.4.1249-1259.2001 . 11158311 PMC99578

[30] Kim H, Rafiuddin-Shah M, Tu HC, Jeffers JR, Zambetti GP, Hsieh JJD, et al Hierarchical regulation of mitochondrion-dependent apoptosis by BCL-2 subfamilies. Nat Cell Biol. 2006;8(12):1348–U19. 10.1038/ncb1499 . 17115033

[31] Antonsson B. Bax and other pro-apoptotic Bcl-2 family "killer-proteins" and their victim, the mitochondrion. Cell Tissue Res. 2001;306(3):347–61. 10.1007/s00441-001-0472-0 . 11735035

[32] Ferri KF, Kroemer G. Organelle-specific initiation of cell death pathways. Nat Cell Biol. 2001;31(11):E255–63. 10.1038/ncb1101-e255 11715037

[33] Tagliarino C, Pink JJ, Dubyak GR, Nieminen AL, Boothman DA. Calcium is a key signaling molecule in beta-lapachone-mediated cell death. J Biol Chem. 2001;276(22):19150–9. 10.1074/jbc.M100730200 . 11279125

[34] Buckley BJ, Whorton AR. Tunicamycin increases intracellular calcium levels in bovine aortic endothelial cells. Am J Physiol-Cell Ph. 1997;273(4):C1298–C305. . 9357774 10.1152/ajpcell.1997.273.4.C1298

[35] Rao RV, Peel A, Logvinova A, del Rio G, Hermel E, Yokota T, et al Coupling endoplasmic reticulum stress to the cell death program: role of the ER chaperone GRP78. Febs Lett. 2002;514(2–3):122–8. 10.1074/jbc.M102225200 . 11943137 PMC3971841

[36] Rizzuto R, Pinton P, Carrington W, Fay FS, Fogarty KE, Lifshitz LM, et al Close contacts with the endoplasmic reticulum as determinants of mitochondrial Ca^2+^ responses. Science. 1998;280(5370):1763–6. 10.1126/science.280.5370.1763 . 9624056

